# Multi-Omics Analysis Reveals Anti-*Staphylococcus aureus* Activity of Actinomycin D Originating from *Streptomyces parvulus*

**DOI:** 10.3390/ijms222212231

**Published:** 2021-11-12

**Authors:** Yuqi Lin, Li Huang, Xiaoyong Zhang, Jiajia Yang, Xiaodan Chen, Fengming Li, Jun Liu, Riming Huang

**Affiliations:** 1 Guangdong Provincial Key Laboratory of Food Quality and Safety, College of Food Science, South China Agricultural University, Guangzhou 510642, China; linyuqi@stu.scau.edu.cn (Y.L.); huangli@stu.scau.edu.cn (L.H.); jiajia_yang2021@163.com (J.Y.); 18125907913@163.com (X.C.); lifm@mail.sustech.edu.cn (F.L.); 2 Joint Laboratory of Guangdong Province and Hong Kong Region on Marine Bioresource Conservation and Exploitation, College of Marine Sciences, South China Agricultural University, Guangzhou 510642, China; zhangxiaoyong@scau.edu.cn; 3 Laboratory of Pathogenic Biology, The Marine Biomedical Research Institute, Dongguan Key Laboratory of Medical Bioactive Molecular Developmental and Translational Research, Guangdong Medical University, Zhanjiang 524023, China

**Keywords:** *Staphylococcus aureus*, *Streptomyces parvulus*, actinomycin D, proteomic and metabolomic profiles

## Abstract

*Staphylococcus aureus* (*S. aureus*) is a common pathogen that causes various serious diseases, including chronic infections. Discovering new antibacterial agents is an important aspect of the pharmaceutical field because of the lack of effective antibacterial drugs. In our research, we found that one anti-*S. aureus* substance is actinomycin D, originating from *Streptomyces parvulus* (*S. parvulus*); then, we further focused on the anti-*S. aureus* ability and the omics profile of *S. aureus* in response to actinomycin D. The results revealed that actinomycin D had a significant inhibitory activity on *S. aureus* with a minimum inhibitory concentration (MIC) of 2 μg/mL and a minimum bactericidal concentration (MBC) of 64 μg/mL. Bacterial reactive oxygen species (ROS) increased 3.5-fold upon treatment with actinomycin D, as was measured with the oxidation-sensitive fluorescent probe DCFH-DA, and H_2_O_2_ increased 3.5 times with treatment by actinomycin D. Proteomics and metabolomics, respectively, identified differentially expressed proteins in control and treatment groups, and the co-mapped correlation network of proteomics and metabolomics annotated five major pathways that were potentially related to disrupting the energy metabolism and oxidative stress of *S. aureus*. All findings contributed to providing new insight into the mechanisms of the anti-*S. aureus* effects of actinomycin D originating from *S. parvulus.*

## 1. Introduction

*Staphylococcus aureus* (*S. aureus*) is a frequent pathogen that causes various serious and chronic infections, for example, osteomyelitis, toxic infective endocarditis, and shock syndrome—which is known as one of the most common reasons for nosocomial and device-related infections [1,2,3]. *S. aureus* infections cause morbidity and mortality in both hospital and community environments, and are found in the human commensal microbiota of the nasal mucosa in 20–40% of the general population [4,5]. *S. aureus* infections lead to a morbidity ranging from 20 to 50 persons/100,000 population, as well as a mortality of 10–30% annually [6]. This accounts for even more deaths than for tuberculosis, viral hepatitis, and acquired immune deficiency syndrome [7,8]. The number and variety of infections caused by these bacteria are gradually increasing in recent years, and so, new antibacterial substances and stronger prevention of bacteria are badly needed.

The progress in medical science has sparked the discovery of powerful antibacterial drugs from microorganisms [9,10]. Among microbes, actinobacteria—especially *Streptomycetes*—have historically been researched for their activities against animal pathogens and plant diseases [11]. Nearly two-thirds of the naturally produced marketed antibiotics have been separated from *Streptomyces* spp. such as alnumycin, fistupyrone, and vinylamycin, but only a small part of it has been explored [12,13]. In the course of the search for new anti-*S. aureus* precursors, the extract of a marine actinobacterium *Streptomyces parvulus* (*S. parvulus*) has shown strong antibacterial activity against multidrug-resistant bacteria [9]. Based on the investigations, it was suggested that actinomycin D is the main secondary metabolite of *S. parvulus*. Although actinomycin D is applied as an anticancer drug, it shows toxicity to human cells and reduces cell viability in a dose-dependent and time-dependent manner [14]; a few investigations of its antibacterial activities have also been carried out [15]. Research has found that actinomycin D from *S. parvulus* has significantly inhibited biofilm formation by all selected *S. aureus* strains [16], but the specific substrates of this strain and its relevant mechanism are still unclear. It is still necessary to obtain more details on the anti-*S. aureus* mechanism of actinomycin D.

Therefore, it is necessary to clarify the modulatory effects of actinomycin D on *S. aureus* by analyzing proteomics and metabolomics, including the identification of observably expressed proteins and metabolites. Consequently, we aimed to find the confirmation of functions of the potential proteins and metabolites in actinomycin D-treated *S. aureus*, and analyzed the pathways regulated by differentially expressed proteins—all this research contributes to the further understanding of the anti-*S. aureus* molecular mechanism of actinomycin D.

## 2. Results

### 2.1. Growth of S. parvulus and Structure of Actinomycin D

**Figure 1:**
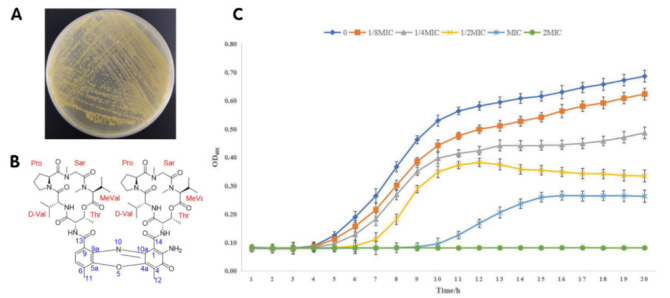
Structural characteristics and anti-*S. aureus* activity of actinomycin D. (**A**) *S. parvulus* cultured on LBA. (**B**) Structural characteristics of actinomycin D. (**C**) Anti-*S. aureus* activity of actinomycin D. OD_600_ represents the optical density under the scanning wavelength of 600 nm.

In Luria-Bertani agar (LBA), the growth of *S. parvulus* could be clearly seen (Figure 1A); after extraction and a high performance liquid chromatography (HPLC) method, the actinomycin D originating from *S. parvulus* was finally obtained. Then, the structure of the actinomycin D was identified by nuclear magnetic resonance (NMR) and mass spectrum (MS) data (Figures S1–S3), and the received molecular structure was further compared with the existing literature [17,18,19]. Thus, the NMR and MS data of actinomycin D was determined as the following: [M+H]^+^, m/z: 1255.6; ^1^H NMR (500 MHz, CD_3_OD): δ 7.54 (d, J = 7.8 Hz, 1H, H-7), 7.47 (d, J = 7.8 Hz, 1H, H-8), 6.18 [d, J = 9.2 Hz, 1H, Pro (α), H-2], 6.12 [d, J = 9.2 Hz, 1H, Pro (β), H-2], 5.25 [dd, J = 6.2, 2.9 Hz, 1H, Thr (α), H-3], 5.19 [dd, J = 6.2, 2.9 Hz, 1H, Thr (β), H-3], 4.82 [d, J = 17.4 Hz, 1H, Sar (α), H-2], 4.72 [d, J = 17.4 Hz, 1H, Sar (β), H-2], 4.71 [d, J = 2.6 Hz, 1H, Thr (α), H-2], 4.67 [d, J = 2.6 Hz, 1H, Thr (β), H-2], 4.09 [m, 1H, D-Val (α), H-3], 4.01 [m, 1H, D-Val (α), H-3], 3.96 [m, 2H, D-Val (α, β), H-2], 3.75–3.02 (m, 5H), 2.97 [m, 6H, 2 × -CH_3_, Me-Val (α, β), N-Me], 2.87 [m, 6H, 2 × -CH_3_, Sar (α, β), N-Me], 2.87–2.59 (m, 5H), 2.59 (s, 3H, -CH_3_, H-12), 2.11 (s, 3H, -CH_3_, H-11), 2.10-1.87 (m, 8H), 1.30 (d, J = 6.1 Hz, 6H, 2 × -CH_3_, Thr (α, β), H-4), 1.15 (d, J = 6.4 Hz, 6H, 2 × -CH_3_, D-Val (α, β), H-5), 0.99 [d, J = 6.4 Hz, 6H, 2 × -CH_3_, Me-Val (α, β), H-4], 0.92 (d, J = 6.9 Hz, 6H, 2 × -CH_3_, D-Val (α, β), H-4), 0.81 [d, J = 6.9 Hz, 6H, 2 × -CH_3_, Me-Val (α, β), H-5]; ^13^C NMR (125 MHz, CD_3_OD): δ 180.4 (C-3), 175.6 [D-Val (α), C-1], 175.4 [D-Val (β), C-1], 175.3 [Pro (α), C-1], 174.9 [Pro (β), C-1], 170.2 [Thr (α), C-1], 170.0 [Thr (β), C-1], 169.9 [2 × C, Me-Val (α, β), C-1], 168.7 [Sar (α), C-1], 168.5 [Sar (β), C-1], 168.3 (C-13), 168.1 (C-13), 149.0 (C-2), 147.1 (C-4a), 146.4 (C-10a), 141.7 (C-5a), 133.8 (C-9), 131.3 (C-7), 130.6 (C-6), 129.1 (C-9a), 126.4 (C-6), 114.2 (C-4), 103.0 (C-3), 76.1 [Thr (β), C-3], 76.0 [Thr (α), C-3], 71.99 [2 × C, Me-Val (α, β), C-2], 59.9 [2 × C, D-Val (α, β), C-2], 58.4 [Pro (α), C-2], 58.3 [Pro (β), C-2], 56.4 [Thr (α), C-2], 56.1 [Thr (β), C-2], 52.5 [2 × C, Sar (α, β), C-2], 48.4 [Pro (α), C-5], 48.1 [Pro (β), C-5], 39.3 [2 × C, Me-Val (α, β), N-C], 35.5 [2 × C, Sar (α, β), N-C], 33.0 [D-Val (α), C-3], 32.8 [D-Val (β), C-3], 32.3 [Pro (α), C-3], 32.1 [Pro (β), C-3], 28.3 [2 × C, Me-Val (α, β), C-3], 23.9 [Pro (α), C-4], 23.7 [Pro (β), C-4], 21.7 [2 × C, Me-Val (α, β), C-5], 19.8 [2 × C, Me-Val (α, β), C-4], 19.6 [2 × C, D-Val (α, β), C-5], 19.3 [2 × C, D-Val (α, β), C-4], 18.1 [Thr (α), C-4], 17.47 [Thr (β), C-4], 15.0 (C-11), 7.59 (C-12) (Figure 1B).

### 2.2. Antimicrobial Activity

Actinomycin D showed an obvious anti-*S. aureus* activity with an MIC value of 2 μg/mL and an MBC value of 64 μg/mL. Our results showed that the growth of *S. aureus* was significantly inhibited by different concentrations of actinomycin D at 8–12 h, and that the growth of bacteria and antibacterial tread at 12–20 h was slower than that at 8–12 h. The above results indicate that the purified product has a continuous antibacterial effect within 20 h (Figure 1C).

### 2.3. Effect of Actinomycin D on Bacterial Morphology

**Figure 2:**
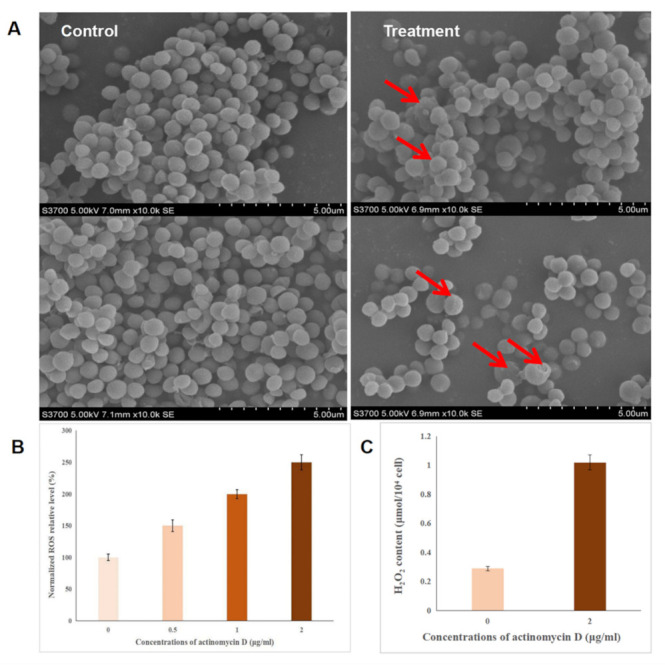
The effect of bacterial morphology and index factors. (**A**) Bacterial morphology affected by actinomycin D in the control group and treatment group. (**B**) Intracellular relative ROS levels in actinomycin D; higher concentrations of actinomycin D with higher ROS relative levels. (**C**) Intracellular relative H_2_O_2_ levels in actinomycin D; the content of H_2_O_2_ in the treatment group (2 ug/mL) was 3.5 times more than the control group.

Aimed at determining the effect of actinomycin D by comparing treated and control groups, SEM analysis was applied to show changes in the morphology of bacteria exposed to actinomycin D. As shown, the control group bacteria showed an intact and smooth membrane with normal morphology, but displayed the leaking of bacterial content in the treatment group, causing significant differences in morphology. As for metabolism, some damaged bacteria could be also seen in the control sample, but this phenomenon was more serious in the treatment sample (Figure 2A).

### 2.4. Intracellular Reactive Oxygen Species (ROS) Detection

ROS is the one of major factors of antibacterial activity, which are produced when antibacterial agents interact with the bacterial solution. We speculated that the ROS level may be induced by actinomycin D and then cause oxidative stress; thus, causing the antibacterial effect. In this research, the ROS levels in bacteria were investigated. An increased fluorescence intensity reflected an increased ROS level—as shown, concentrations of 0.5 ug/mL, 1 ug/mL, and 2 ug/mL actinomycin D gradually showed a higher relative ROS level, with a dose-dependent effect according to the concentration of actinomycin D (Figure 2B). Thus, the antibacterial mechanism of actinomycin D may be associated with increased ROS formation, which shows the increased antibacterial activity of actinomycin D.

### 2.5. The Detection of H_2_O_2_

H_2_O_2_ has been recognized as the major ROS in redox regulation of biological activities [20,21,22], and is also a versatile pleiotropic physiological signaling agent. In our research, the effects of actinomycin D on H_2_O_2_ levels were detected by the hydrogen peroxide kit—the results showed that the H_2_O_2_ in the control group was 0.29 μmol/10^4^ cells and the H_2_O_2_ in the treatment group was 1.02 μmol/10^4^ cell. The content of H_2_O_2_ in the treatment group was 3.5 times higher than the control group; this indicated that the expression of catalase may be inhibited by actinomycin D, which causes the accumulation of H_2_O_2_ (Figure 2C).

### 2.6. Proteomics Profile of S.aureus Treated with Actinomycin D

#### 2.6.1. Label-Free Proteomics-Based Protein Identification

**Figure 3:**
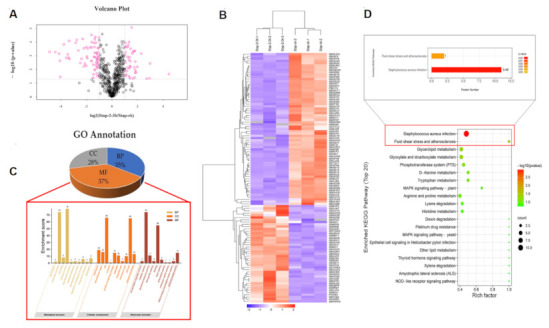
Proteomics results of *S.aureus* treated with actinomycin D. (**A**) Volcano plot of DEPs, Stap-2–3 h represents the treatment group, Stap-ck represents the control group, and DEPs (Regulated > 2.0-fold; *p* < 0.05) are represented by the pink color. (**B**) Hierarchical cluster analysis of all the DEPs. (**C**) Gene ontology annotation of differentially expressed proteins. BP represents the biological processes, MF represents molecular function and CC represents the cellular components of proteins; the top 10 enrichments of BP, CC, and MF are shown. (**D**) Top 20 KEGG pathways of the identified DEPs. Color represents *p*-value; the red color represents low *p*-value, the green color represents high *p*-value; the pathways with a red color represent those that are altered and the circle size represents gene number.

A label-free proteomics method was used for the quantitative proteomic analysis of the two groups (the group treated with 0.125 µg/mL actinomycin D and the control group). All the differentially expressed proteins (DEPs) with a fold-change value over 2 and under 0.5 are listed in Table S1. The volcano plot shows a total of 128 DEPs in the two groups (Figure 3A). Proteins with significant changes in their expression were detected, and 110 proteins (43 up-regulated proteins and 67 down-regulated proteins) were enriched in the actinomycin D-treated group. Meanwhile, 5 newly emerged proteins were identified in the treatment group, and 13 proteins were solely observed in the control group. The analysis of hierarchical clusters showed that all of the DEPs clustered into two groups; the DEPs in the treatment group did not cluster together with those in the control group (Figure 3B). The above analysis indicates that there is a significant difference in the proteins between the control and treatment groups; thus, further investigation is necessary.

#### 2.6.2. Gene Ontology (GO) Annotation

Gene ontology (GO) annotation provides a helpful way to systematically analyze the properties of target proteins, and is used to study the function of DEPs [23,24]. GO annotation was further divided into three components: molecular function (MF), cellular components (CC), and biological processes (BP). The GO analysis showed that the 128 DEPs were subdivided into 30 hierarchical GO classifications. The classification of identified DEPs linked with MF, BP, and CC was 37%, 35%, and 28%, respectively, and all the essential processes of BP, MF, and CC are listed as visualized diagrams (Figure 3C). We concluded that the classification of the GO annotation showed that the BP was highly enriched in cellular processes (37%) and metabolic processes (34%), and that at the molecular function level, 48% DEPs were involved in catalytic activity and 36% were associated with binding—while most of the DEPs were ontologically related to the cell (32%), cell part (32%), protein-containing complex (7%), nucleoid membrane (9%), extracellular region (8%), organelle (5%), membrane part (5%), and organelle part (1%) of the cellular components. These results revealed that many proteins in *S.aureus* under the treatment of actinomycin D might participate in metabolism and cellular processes, which are located in different cell parts and organelles.

#### 2.6.3. Analysis of Main Metabolic Pathways

Many proteins are vital enzymes in metabolic pathways. To illuminate the key metabolic pathways and enzymes that are essential in the treatment group, the KEGG database and Blast2go software were employed. The KEGG results indicated that a total of 128 DEPs participated in 76 signaling pathways, revealing that the top 20 metabolic pathways were *S. aureus* infection, fluid shear stress and atherosclerosis, glycerolipid metabolism, glyoxylate and dicarboxylate metabolism, the phosphotransferase system (PTS), D-Alanine metabolism, arginine and proline metabolism, lysine degradation, histidine metabolism, tryptophan metabolism, ribosome and pyrimidine metabolism, the citrate cycle (TCA cycle), arginine biosynthesis, pyruvate metabolism and vancomycin resistance (Figure 3D).

### 2.7. Metabolites Data of S.aureus Treated with Actinomycin D

#### 2.7.1. Analysis of Main Metabolites

**Figure 4:**
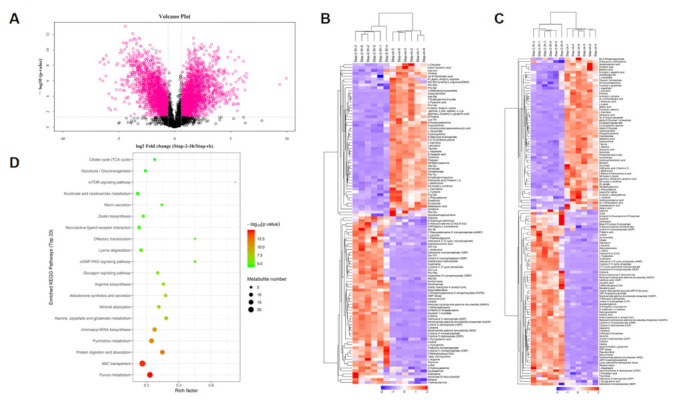
Metabolites results of *S.aureus* treated with actinomycin D. (**A**) Volcano plot of significantly differently metabolites. (**B**) Hierarchical cluster heat map of differentiated metabolites in positive mode. (**C**) Hierarchical cluster heat map of differentiated metabolites in negative mode. (**D**) Top 20 pathways encompassed by the differentiated metabolites. Color represents *p*-value; a red color represents low *p*-value, a green color represents high *p*-value; the pathways showing a red color are significantly altered, and the circle size represents the gene number.

According to the OPLS-DA model for choosing the main metabolites, with one of the screening criteria being the *p*-value, the metabolites with a *p*-value over 0.05 and under 0.1 (0.05 < *p*-value < 0.1) were considered as the differentiated metabolites. After the data was analyzed and processed, the biologically different metabolites were selected. The results showed that the metabolites were significantly different between the treatment and control group; the rationality of the identified metabolites was evaluated (Figure 4A). Hierarchical cluster analysis was used to show the changes in metabolites between the treatment group and control group of *S. aureus* based on the degree of difference or similarity between them. The analysis was conducive to studying changes in candidate metabolic processes and the accurate selection of target metabolites; the analysis graph revealed that metabolite differences within the groups were rare, and that the differences in metabolites between the two groups were obvious—which indicated that the results of the metabolite detection and identification were reasonable and reliable (Figure 4B,C). As shown, 190 significantly changed metabolites were identified, and in the treatment group, 91 metabolites were significantly down-regulated, while 99 metabolites were significantly up-regulated (Table S2).

#### 2.7.2. Analysis of the Main Metabolic Pathways

KEGG enrichment analysis was performed on the differentiated metabolites, and 90 signal pathways were enriched (*p* < 0.05). The pathways involved in amino acid metabolism in these pathways were “Lysine degradation”, “Lysine biosynthesis”, “Arginine and proline metabolism”, “Arginine and proline metabolism”, “Alanine, aspartate, and glutamate metabolism”, “Glycine, serine and threonine metabolism”, “D-Alanine metabolism”, “Valine, leucine, and isoleucine biosynthesis”. The pathways involved in carbohydrate metabolism included “Glyoxylate and dicarboxylate metabolism”, “Citrate cycle”, “Glycolysis/Gluconeogenesis” and so on. The pathways involved in energy metabolism included “Carbon fixation pathways in prokaryotes”, “Methane metabolism”, and “Oxidative phosphorylation”. The top 20 pathways with lower *p*-values were selected to draw the KEGG enrichment histogram, as shown in the diagram; different metabolites were mainly related to “Alanine, aspartate, and glutamate metabolism”, including L-alanine, succinate, L-aspartate, L-glutamate, N-acetyl-L-aspartic acid, citrate, L-asparagine, argininosuccinic acid, D-aspartic acid, and different metabolites participating in the “Citrate cycle” pathway included succinate, cis-aconitate, citrate, acetyl coenzyme A, and phosphoenolpyruvate (Figure 4D).

### 2.8. Proteomics–Metabolomics Correlation Network in S.aureus Treated with Actinomycin D

**Figure 5:**
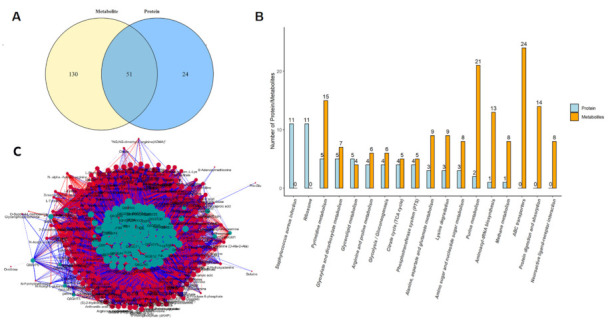
Proteomics–metabolomics correlation network in *S.aureus* treated with actinomycin D. (**A**) Venn diagram of the differentiated proteins and metabolites. (**B**) Top 18 pathways with the largest number of differentiated proteins and metabolites. (**C**) Network of the differentiated proteins and metabolites. Blue represents proteins, orange represents metabolites, the abscissa text represents the name of the pathway, the ordinate represents the number of differential proteins and metabolites involved.

According to the changes of proteins and metabolites, metabolic pathways were chosen as carriers and a mapping analysis was performed to compare the data from the proteome and the metabolome. Fifty-one common targets between proteins and metabolites were recognized, and the top 18 pathways with the largest total number of differentiated proteins and metabolites were enriched (Figure 5A,B). Additionally, correlation network analysis was also employed to find new insights between differentiated proteins and metabolites (Figure 5C).

## 3. Discussion

*S.aureus* is common in living environments, such as the air and sewage; they are also recognized as a common food-borne pathogenic bacteria, which is commonly parasitic in the skin, nasal cavity, throat, stomach, carbuncle, and suppurative wounds of people and animals [25]. *S.aureus* has been regarded as the most common cause of hospital-acquired infection, causing clinical disease in 2% of all patient admissions; however, the incidence of multidrug-resistant *S.aureus* isolates has increased globally as research has deepened into its inhibiting drugs—for example, the methicillin-resistant *S.aureus* (MRSA) [26]. Thus, research is still focused on inhibiting *S.aureus,* due to its serious influence on the living environment, human health, and ecological systems. More and more methods of combating *S. aureus* have been emphasized; for example, Kaixiang Zhou et al. found that nanoparticles contribute to inhibiting the formation of biofilms and enhancing intracellular delivery of antibiotics, thus enhancing their activity against *S. aureus* [27]. Another new study has also found the same action against *S. aureus;* as the research shows, the recombinant HtrA efficiently suppressed in vitro biofilm formation by clinical isolates of *S. aureus* [28]. Otherwise, the study carried out by Irshad S. Sharafutdinov et al. found that the sulfonyl derivative of 2(5H)-furanone against planktonic and biofilm-associated methicillin-resistant and -susceptible *S.aureus* led to antimicrobial effects [29]; moreover, Cin Kong et al. concluded that the benzimidazole derivative, UM-C162, contributes to the suppression of *S.aureus* [30]. However, actinomycin D is well-known as an antibiotic and has been widely focused on. In this work, we aimed to ensure that the actinomycin D originating from *S.parvulus* contributes to inhibition of *S.aureus* after its extraction and fermentation, and finally, its potential mechanisms and relevant pathways were analyzed. In the obtained data, we found that ROS and H_2_O_2_ have a dose-dependent effect on the content of actinomycin D; it has been reported that changes of ROS in bacteria are mainly caused by the autoxidation of NADH dehydrogenase II in the respiratory chain [31]. Of the differentially expressed proteins, catalase is related to stress response [14]. The ROS levels in the two groups were analyzed by cell fluorescence intensity—we found that the expression levels of catalase (Q6GH72) were reduced when cells were treated by actinomycin D. The role of the protein in protecting cells from oxidative damage was deduced by analyzing the ROS levels under the treatment of actinomycin D. Otherwise, aconitate hydratase A (Q6GH55; AH), related to the TCA cycle, influenced the level of free fatty acids. It is known that free fatty acids provide energy for the uncoupling of respiration and phosphorylation in mitochondria, which can reduce the extent of ROS production at high membrane potentials and subsequent high ratios of ATP/ADP and NADH/NAD+ as needed [14]. All the mentioned reactions suggest that the changes in ROS may be caused by the down-regulation of AH and catalase. The observed increases in ROS suggest that ROS might generate more NADPH via isocitrate dehydrogenase and the pyruvate–malate cycle through stimulation of the cells. As a matter of fact, as a result of electron leakage during respiration, the components of the electron transport chain will be damaged during the production of ROS, and a suitable amount of ROS could facilitate lipid accumulation [15,32,33]. Otherwise, ROS generated by nitrogen starvation could also provide neutral lipid accumulation in Chlorella cells, and increased oxidative stress through addition of H_2_O_2_ could induce the accumulation of neutral lipids. In our research, we found that the content of H_2_O_2_ was increased in the treatment group. Consequently, the increased mitochondrial oxidative stress in the actinomycin D-treated *S. aureus* likely produced additional ROS and H_2_O_2_, thus stimulating many cellular compounds, such as unsaturated fatty acids. Otherwise, the down-regulated DEPs are mostly related to carbohydrate metabolic pathways, such as the tricarboxylic acid cycle (TCA cycle), glycolysis/gluconeogenesis, pyruvate metabolism, starch and sucrose metabolism, and the pentose phosphate pathway; all these changes imply that carbohydrate metabolism may be suppressed by actinomycin D. It is known that carbohydrate metabolism responds to supply substrates and energy to biological processes; the inhibition of carbohydrate metabolism implies limitations of the energy supply of the cell [34]. The TCA cycle is regarded as the pivotal metabolic pathway for supplying energy, and pyruvate is the precursor for the TCA cycle—so decreases in pyruvate are a key factor in TCA cycle inactivation [35]. Enzymes related to the TCA cycle and pyruvate metabolism, such as aconitate hydratase A (Q6GH55; ACNA), 2-oxoglutarate dehydrogenase E1 component (Q6GGZ5; ODHA), and malate: quinone oxidoreductase 1 (Q6GE66; MQO1), were all down-regulated in the actinomycin D treatment group. On the one hand, ACNA, related to the TCA cycle, has been identified, which could catalyze the transformation of methyl-cis-aconitate into D-three-α-methyl isocitrate—which not only inhibits the production of NADP-isocitrate dehydrogenase, but also catalyzes the reaction of reversible isomerization of citrate to isocitrate; moreover, the reaction catalyzed by ACNA is the preliminary step of the TCA cycle [36]. On the other hand, ODHA is a rate-limiting enzyme in the TCA cycle and produces succinyl-CoA by oxidative decarboxylation of 2-oxoglutarate, and MQO1 is involved in the TCA cycle and pyruvate metabolism—catalyzing the oxidation of malate to oxaloacetate, and in the meantime, reduction of ubiquinone to ubiquinol [37]. In general, decreases in the levels or activity of these enzymes will suppress the production of ATP. Acetyl-CoA is an important intermediate metabolite in the metabolism of energy substances—the three major nutrients of sugar, fat, and protein converge through it into the TCA cycle. Under this process, nutrients are completely oxidized to produce carbon dioxide and water, and finally, to release energy for synthesizing ATP. The omics results showed that the content of acetyl-CoA, citric acid, and cis-aconitic acid increased, and the content of succinic acid decreased, so we speculated that actinomycin D inhibits the growth and reproduction of *S.aureus* mainly by suppressing the expression of ACNA, ODHA, and MQO1, which leads the stagnation of energy transformation. In addition, 4,4-diaponeurosporen-aldehyde dehydrogenase (Q2FWX9; AldH) and glyceraldehyde-3-phosphate dehydrogenase (Q6GG19; GapA) were also inhibited. AldH is related to glycolysis, which involves the oxidation of 4,4-diaponeurosporen-4-al into 4,4-diaponeurosporenoic acid in the staphyloxanthin biosynthetic pathway in *S. aureus*; as the 6th enzyme in the staphyloxanthin biosynthetic pathway of S. aureus, it is necessary for staphyloxanthin formation [38]. Another enzyme, GapA is involved in the glycolysis step in which ATP is expressed, during the conversion of 1,3-bisphosphoglycerate to 3-phosphoglycerate [39]. Owing to the down-regulated expression of AldH and GapA, the production of ATP from glycolysis was inhibited.

In this study, most of the amino acid metabolism enzymes, such as 3-dehydroquinate dehydratase (Q8NXI0), 1-pyrroline-5-carboxylate dehydrogenase (Q8NUR2), argininosuccinate synthase (Q6GIC7), argininosuccinate lyase (Q8NXF3), 4,4-diaponeurosporen-aldehyde dehydrogenase (Q2FWX9), aminomethyltransferase (Q6GGG20), glycine cleavage system H protein (Q6GII3), ornithine aminotransferase 2 (Q6GAW9), 2-oxoglutarate dehydrogenase E1 component (Q6GGZ5), dihydrolipoyllysine-residue succinyltransferase component of 2-oxoglutarate dehydrogenase complex (Q6GGZ6), formimidoylglutamase (Q6GEA1), and urocanate hydratase (Q6GEA4) were down-regulated, and all the proteins related to proline metabolism were inhibited. For example, in an NAD+-dependent process, the conversion of 1-pyrroline-5-carboxylate back to glutamate is the second step in proline degradation; this molecule is a restriction enzyme of the mitochondria matrix that is vital in the anaplerotic role of proline liberated from proteins. Glutamate is the product of this, and can enter the TCA Cycle as alpha-KG—therefore allowing proline, either released by matrix metalloproteinases or by the import of free proline, to be used for anaplerosis and energy. The enzyme 1-Pyrroline-5-carboxylate dehydrogenase may play a significant role as an exit point for intermediates from the proline cycle [40]. The down-regulation of 1-Pyrroline-5-carboxylate dehydrogenase might be related to the inhibition of the TCA cycle and the oxidative decarboxylation of isocitrate, which would reduce the production of energy and the supplementation of NADP. Additionally, arginosuccinate synthetase catalyzes the transformation of aspartate into arginine, which serves as the penultimate step of arginine biosynthesis [41], all of this information suggests that protein synthesis was inhibited during treatment with actinomycin D.

It has been reported that amino acids are released by protein degradation for respiration when carbohydrate metabolism is inhibited [42]. Hence, amino acid metabolism disruption might be an important compensatory mechanism in *S. aureus* when treated with actinomycin D. According to our metabolomics results, glutamate, a significant substance in cell wall biosynthesis, was decreased in the treatment group; it has been reported that glutamate is not only one of the amino acids in the pentapeptide component of the cell wall, but is also the monomer of poly-γ-glutamate [35], which is also expressed on the surface of the *S. aureus* cell wall. Furthermore, research has pointed out that inhibiting the activity of glutamine synthase in mycobacteria could reduce poly-γ-glutamate levels and prevent the replication of bacteria [43]. Consequently, we speculated that disruption of the glutamate biosynthetic pathway may be detrimental to cell wall synthesis. This is consistent with the results of the SEM, as the cells ruptured after actinomycin D treatment. Under stress from actinomycin D, it may become harder for the cell to keep its wall integrity or to form a new wall to accomplish cell replication. Furthermore, there may be another aspect—glutamate potentiates the effects of antibiotics that target the ribosome, so a decrease in glutamate within the cell could also be a pathway to enable (at least a subset of the cells) to survive this stress; otherwise, the degradation of some amino acids, such as valine, leucine and isoleucine degradation, might affect lipid synthesis, as it could reduce levels of acetyl-CoA substrate [44]. In summary, we deduced that actinomycin D inhibited the growth and reproduction of *S. aureus* by affecting proteins (such as the mentioned enzymes) related to oxidative stress and energy metabolism. However, all the mentioned facets of this potential mechanism still need further investigation.

## 4. Materials and Methods

### 4.1. Isolation and Identificationof Actinomycin D

We isolated an SCAU-062 strain from a coral sample from the South China Sea (110°40′E, 15°20′N; Guangzhou, China), which was further identified as *Streptomyces* sp., with a 99.8% similarity with the strain *S. parvulus* OUCMDZ-2554 (KF985960) through the GenBank database [45]. After growing the bacteria, we isolated a crude extract, which was subjected to a silica gel using a gradient of petroleum ether/acetone (from 7:3 to 0:1) (Sigma, Shanghai, China) to obtain eight fractions (A−H). Fraction C was further separated by semi-preparative reversed-phase HPLC (Waters Prep C18 column, 150 × 19 mm, 5 μm, 2 mL/min) (Waters, Milford, MA, USA) to obtain purified actinomycin D (45% MeOH/H_2_O, tR = 24.1 min, 156 mg), which was identified as actinomycin D by NMR spectra recorded on a Bruker AV 600 MHz NMR spectrometer (Bruker, Bremen, Germany) and MS data performed on an AB SCIEX (Boston, MA, USA) MS spectrometer.

### 4.2. Fermentation and Growth of S. aureus

In the American Type Culture Collection (ATCC), the *S. aureus* strain ATCC 25,923 is obtained. It was cultured on LBA (HuanKai, Guangzhou, China) at 37 °C, which included peptone 10 g/L, yeast extract 5 g/L, sodium chloride 5 g/L, agar 15 g/L, and dextrose 1 g/L. The fermentation medium was a luria-bertani broth liquid medium. We used an inoculating loop to transfer a small number of *S. aureus* from a seed tube (30% glycerol, −80 °C) to the LB broth medium, then cultivated it in an electrothermal incubator at 37 °C. The LB broth medium surface was covered with thallus and cut into square pieces of 1 cm × 1 cm by using a sterile knife after 1–2 days of cultivation. The square pieces of the cell were transferred into 1000 mL flasks containing 200 mL of LB broth medium.

### 4.3. MIC and MBC of Actinomycin D

Through the broth microdilution method, the MIC of the purified compound was measured. Different concentrations of the purified compound of 0.25 μg/mL, 0.5 μg/mL, 1 μg/mL, 2 μg/mL, 4 μg/mL, 8 μg/mL, 16 μg/mL and 32 μg/mL were added to the *S. aureus*, and grown to the logarithmic period (0.3–0.5 OD440) [46]. *S. aureus* cultured in Mueller Hinton Broth (MHB, 100 μL, HuanKai, Guangzhou, China) and then mixed with 100 μL of different concentrations of actinomycin D, a control group containing 100 μL of the test compound at different concentrations and 100 μL of MHB, a positive control consisting of 100 μL of bacterial culture and 100 μL of MHB, and a negative control containing only 200 μL of MHB, and the 96-well plates were incubated at 37 °C; then, the OD value was measured at 440 nm after 24 h. The lowest concentration of the tested compound that inhibited the growth of bacteria was defined as the MIC. After the MIC value of actinomycin D was determined, the wells in the 96-well plate without bacterial growth were selected, and 10 μL of the liquid was taken from the wells on the MH agar plate and incubated at 37 °C for 20 h. The concentration corresponding to the agar plate (HuanKai, Guangzhou, China) where no bacterial growth was observed was the MBC.

### 4.4. Growth Curve of S. aureus Affected by Actinomycin D

The growth curve was detected by the automatic growth curve analyzer (Bioscreen C, Oy Growth curves Ab Ltd., Helsinki, Finland), and the susceptibility curve of actinomycin D to *S. aureus* was determined by the micro broth dilution method. 10 μL of the bacterial suspension (106 CFU/mL) of *S. aureus* was cultured in a 96-well plate containing 180 μL MH broth medium, and 10 μL of different concentrations of actinomycin D (0, 1/8 MIC, 1/4 MIC, 1/2 MIC, MIC, 2 MIC) was added to each well. The blank control group had only 200 μL of MH broth. An automatic growth curve analyzer was used to continuously detect OD600 at 37 °C for 24 h, and then the growth curves were drawn and processed at different MIC values, according to the absorbance value.

### 4.5. Scanning Electron Microscopy (SEM) Analysis

Comparing the common methods for showing the effects of antibiotics on membranes, confocal laser scanning microscopy [5] and scanning electron microscopy were the most recommended, and a scanning electron microscope (Zeiss, Oberkochen, Germany) was finally decided on to analyze the bacterial morphology. *S. aureus* was cultured in 100 mL LB broth medium for 24 h. The treatment group had actinomycin D added to make a final concentration half of the MIC value. The cells were collected by centrifugation and washed 3 times with 0.1 M PBS. The cells were fixed in 2.5% glutaraldehyde (PBS buffer solution). The sample was placed in a refrigerator at 4 °C for 5–10 min and centrifuged for 10 min (4000 r/min; Sorvall^TM^ LYNX, Menlo Park, CA, USA) to remove the supernatant, then fixed with glutaraldehyde fixative solution at 4 °C for 15–30 min. The cells were washed with PBS 3–5 times, then the bacterial sample was fixed with 1% osmium acid for 30 min. The sample was thoroughly cleaned for more than 30 min, and then washed twice with 50%, 70%, 80%, 90%, and 100% ethanol—each for 30 min. Isoamyl acetate was used to replace the dehydrating agent and residual moisture in the sample, and then conventional carbon dioxide critical point drying was performed. The stereo microscope was used to observe the sample and confirm the observation surface. Conductive glue was used to paste the sample onto the sample holder, and to spray the metal. A Hitachi s-3700 n tungsten filament scanning electron microscope (Zeiss, Oberkochen, Germany) was used to observe and photograph the morphological changes of the bacteria.

### 4.6. Intracellular Detection of Reactive Oxygen Species (ROS)

2,7-Dichlorofluoresce diacetate (DCFH-DA) is an oxidation-sensitive fluorescent probe for measuring ROS. For our study, *S. aureus* was incubated to 108 CFU/mL, then washed thrice by PBS. DCFH-DA was mixed with the culture medium at a ratio of 1:1000, and the mixture was cultured for 30 min at 37 °C. Then, the bacteria were gathered by centrifugation and washed twice to detach the DCFH. The cleaned cells were treated with actinomycin D at increasing concentrations from 0μg/mL to 2 μg/mL. A fluorescence spectrophotometer (Thermo Scientific, Waltham, MA, USA) was used to measure the fluorescence intensity of DCF with an emission wavelength of 525 nm and excitation wavelength of 488 nm [47].

### 4.7. Detection of H_2_O_2_

*S. aureus* were cultivated to the logarithmic phase, then 2% of the inoculum was added to LB liquid medium. Actinomycin D was added to the inoculum to make the medium reach the MIC value in the treatment group. The cell was cultivated at 37 °C at 150 r/min for 24 h. The bacteria were gathered in a centrifuge tube, and the supernatant was discarded after centrifugation. One mL of extraction solution was added for 5 million bacteria, the bacteria were ultrasonicated (power 20%, ultrasonic 3 s, interval 10 s, repeat 30 times). The cell were then centrifuged at 8000× *g*, 4 °C for 10 min, then the supernatant was taken (Beckman Coulter, Brea, CA, USA), and tested on ice. A hydrogen peroxide content detection kit (Solarbio, Corning, NY, USA) was used for the determination.

### 4.8. Label-Free Quantitative Proteomics

Based on the method of label-free quantitative proteomics [34], the cells were centrifuged at 5000× *g* for 3 min, then washed by phosphate-buffered saline (PBS) thrice. The cells were mixed with 200 μL of lysis buffer (100 mM DTT, 4% SDS, pH 8.0, 150 mM TrisHCl) on ice. Homogenizer (Fastprep-24^®^, MP Biomedical) was used to break the cells, and the lysates were boiled for 5 min. Then, the samples were homogenized by ultra-sonication and boiled for 5 min. Undissolved cellular debris was centrifuged at 14,000 rpm for 15 min and the supernatants were collected for further investigation. The protein concentration of the supernatants was measured by a BCA Protein Assay Kit (Bio-Rad, Hercules, CA, USA). Based on the reported filter-aided sample preparation (FASP) procedure, the digestion of protein (250 μg for each sample) was carried out [48]. C18 Cartridges (Sigma, Burlington, MA, USA) were used to desalt the peptides in each sample, vacuum centrifugation was used for concentrating the samples, and 40 μL of 0.1% (*v*/*v*) trifluoroacetic acid was added to reconstitute the samples. MS experiments were performed on a Q Exactive mass spectrometer coupled to an Easy nLC (Thermo Fisher Scientific, Waltham, MA, USA).

### 4.9. Untargeted Metabolomics Methods

400 μL of acetonitrile/methanol (1:1, *v*/*v*, 4 °C) were mixed with 100 μL aliquots to eliminate the proteins in the samples at 4 °C. The mixture was centrifuged (14,000× *g*, 4 °C) for 15 min, and then the supernatant was desiccated in a centrifuge. The cells were re-dissolved in 100 μL water/acetonitrile (1:1, *v*/*v*) solvent for LC–MS analysis. Quality control (QC) samples were produced by mixing 10 μL of every single sample together to control for the reproducibility of the instrumental measurements. The QC samples were evenly inserted and at every 5 samples. A UHPLC (1290 Infinity LC, Agilent Technologies) and a quadrupole time-of-flight mass spectrometer (AB Sciex TripleTOF 6600) were employed for the analysis. An ACQUIY UPLC BEH column (2.1 mm × 100 mm, 1.7 µm, waters, Ireland) was used for separation of the samples. Twenty-five mM of ammonium hydroxide and 25 mM of ammonium acetate in water (A) and acetonitrile (B) were used as the mobile phases in both ESI positive and negative modes. The gradient conditions were: 15% A maintained for 1 min, increased to 35% over 11 min, gradually increased to 60% over 0.1 min and retained for 4 min, reduced back to 15% over 0.1 min, and finally, a 5 min equilibration.

### 4.10. Statistical Data Analysis

The MS data were processed by MaxQuant software. For annotation of adducts and isotopes, the Collection of Algorithms of MEtabolite pRofile Annotation (CAMERA) was used. By comparing MS/MS spectra and accuracy m/z values (<25 ppm) with an internal database built with available authentic standards, a compound evaluation of metabolites was conducted. To show its contribution to the classification, the variable importance in projection (VIP) value of each variable was computed in the OPLS-DA model. To measure the significance of each protein and metabolite, proteins and metabolites in which the VIP exceeded 1.0 were further evaluated by Student’s t-test at a univariate level (*p*-values < 0.05 were regarded as statistically meaningful), and the KEGG pathways enrichments, and all differentially expressed proteins/modified peptides/genes and metabolites, were queried and mapped to pathways based on the online Kyoto Encyclopedia of Genes and Genomes (KEGG, http://www.kegg.jp/, 15 September 2021). Enrichment analysis was also performed. R Version 3.5.1 was used to combine the KEGG annotation and enrichment results of the two omics. A Venn diagram and bar plot were drawn. Both the GO terms and KEGG pathways were chosen according to the *p*-value (<0.05); the terms with lower *p*-values were recognized as the more credible ones.

## 5. Conclusions

In summary, the present investigation showed that actinomycin D isolated from a marine *S. parvulus* had a significant anti-*S. aureus* effect; in our work, we found that the MIC and MBC values of anti-*S. aureus* were 2 μg/mL and 64 μg/mL, respectively. Otherwise, ROS generated by actinomycin D under different concentrations were measured with the oxidation-sensitive fluorescent probe DCFH-DA, and H_2_O_2_ increased 3.5 times with treatment by actinomycin D. In addition, the analysis of proteomics identified 640 proteins in the treatment group and the control group; 128 DEPs were recognized, including 43 up-regulated proteins and 67 down-regulated proteins, 5 proteins specific to the treatment group, and 13 proteins specific to the control group. The analysis of metabolomics identified 190 significantly different metabolites in the two groups, including 99 up-regulated metabolites and 91 down-regulated metabolites. The results of the GO enrichment and KEGG pathway analysis indicated that carbohydrate metabolism, amino acid metabolism, and energy generation biosynthesis were suppressed, whereas the ribosome was active. Based on the above analyses, we speculated that actinomycin D might inhibit *S. aureus* by disrupting these metabolic processes—mainly emphasizing oxidative stress and energy metabolism. However, further research is still required to obtain a deeper understanding of these mentioned processes.

## Supplementary Materials

The following are available online at https://www.mdpi.com/article/10.3390/ijms222212231/s1.
