# Supplementary material for: Multi-Omics Analysis Reveals Anti-Staphylococcus aureus Activity of Actinomycin D Originating from Streptomyces parvulus
Source: Int J Mol Sci. 2021 Nov 12;22(22):12231. doi: 10.3390/ijms222212231 (PMC8621895; doi:10.3390/ijms222212231)
Supplement: Supplementary file 1 [file ijms-22-12231-s001.zip › ijms-1416487-supplementary.pdf]

Supplementary Material

Supplementary Figures

Figure S1: ^{1}H NMR spectrum of actinomycin D

Figure S2: ^{13}C NMR spectrum of actinomycin D

Figure S3: MS data of actinomycin

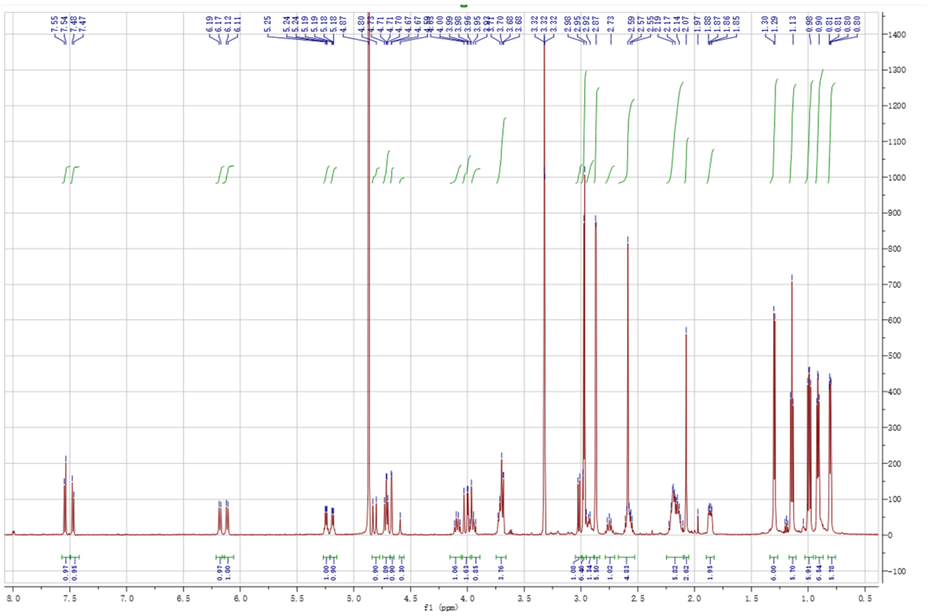

Figure S1. ^{1}H NMR spectrum of actinomycin D

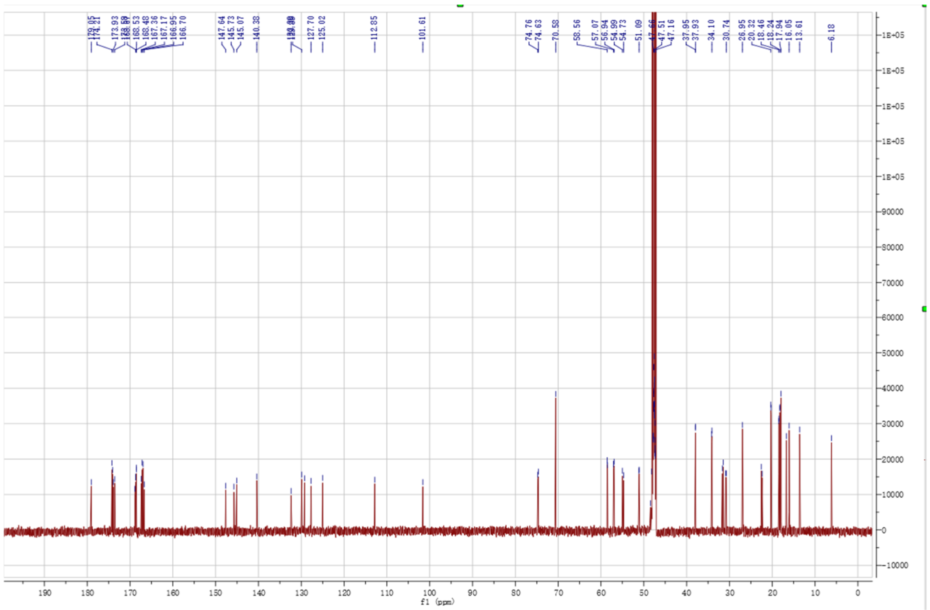

Figure S2. ^{13}C NMR spectrum of actinomycin D

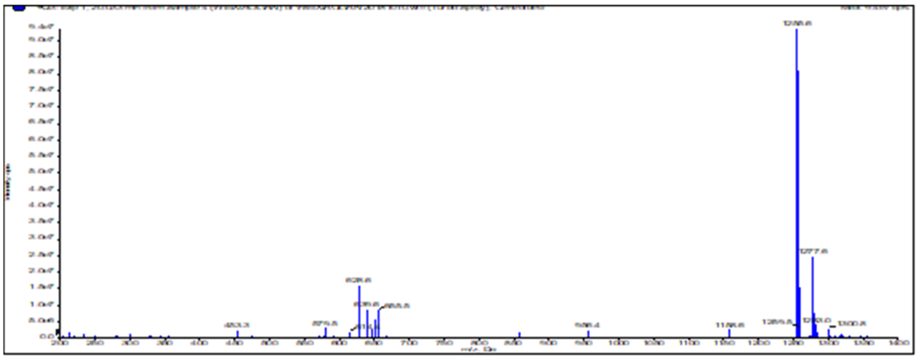

Figure S3. MS data of actinomycin D

Supplementary Tables

Table S1: The DEPs in treatment group and control group

Table S2: The differently changed metabolites in treatment group and control group

Table S1. The DEPs in treatment group and control group.

Protein	Protein Name	Gene Name	Fold Changes [1]	p-value	-log(p-value)	GO Category
Q5HJZ6	Plasmid recombination enzyme type 3	*pre*	21.98	0.0217	1.66	CC BP MF
Q6G8P5	Translation initiation factor IF-3	*infC*	7.85	0.0036	2.45	CC MF
Q6GJA7	Serine-aspartate repeat-containing protein C	*sdrC*	7.63	0.0443	1.35	CC BP
Q8NWZ5	Isoprenyl transferase	*uppS*	6.53	0.0464	1.33	MF
Q99RY1	Urease accessory protein UreE	*ureE*	4.92	0.0007	3.16	CC BP MF
Q6GGC5	30S ribosomal protein S21	*rpsU*	3.96	0.0064	2.19	CC BP MF
Q8NYR1	Deoxyribose-phosphate aldolase 1	*deoC1*	3.62	0.0160	1.80	CC BP MF
Q8NWN3	Chorismate synthase	*aroC*	3.54	0.0027	2.58	BP MF
Q6GEE4	Urease subunit alpha	*ureC*	3.53	0.0112	1.95	CC BP MF
Q5HEB7	D-alanine--D-alanine ligase	*ddl*	3.53	0.0173	1.76	CC BP MF
Q2FUW3	Accessory Sec system protein Asp1	*asp1*	3.47	0.0005	3.34	BP
Q6GI01	Phosphoenolpyruvate-protein phosphotransferase	*ptsI*	3.42	0.0391	1.41	CC BP MF
Q2YWE5	4,4-diaponeurosporene oxygenase	*crtP*	3.36	0.0070	2.15	BP MF
Q6GF42	10 kDa chaperonin	*groS*	3.27	0.0004	3.35	CC BP MF
Q6GEK2	50S ribosomal protein L15	*rplO*	3.12	0.0005	3.28	CC BP MF
Q6GHT5	Glutamate racemase	*murI*	3.09	0.0462	1.34	BP MF
Q99U54	Extracellular matrix-binding protein EbhA	*ebhA*	3.00	0.0007	3.17	CC BP
Q6GDN1	Probable transglycosylase IsaA	*isaA*	2.90	0.0000	5.07	CC BP MF
Q6GJF0	Pyridoxal 5-phosphate synthase subunit PdxS	*pdxS*	2.76	0.0003	3.52	BP MF
Q6GJD1	50S ribosomal protein L11	*rplK*	2.71	0.0025	2.60	CC BP MF
Q6GH52	Glycerol-3-phosphate acyltransferase	*plsY*	2.70	0.0063	2.20	CC BP MF
Q99WZ6	Pyruvate formate-lyase-activating enzyme	*pflA*	2.67	0.0438	1.36	CC BP MF
Q931Q3	50S ribosomal protein L27	*rpmA*	2.66	0.0056	2.25	CC BP MF

Q99VP5	NADPH-dependent 7-cyano-7-deazaguanine reductase	*queF*	2.65	0.0427	1.37	CC BP MF
Q99R97	PTS system glucoside-specific EIICBA component	*glcB*	2.59	0.0013	2.90	CC BP MF
P03064	Replication initiation protein	*repC*	2.56	0.0083	2.08	BP MF
Q6GEK1	50S ribosomal protein L30	*rpmD*	2.52	0.0001	4.00	CC BP MF
Q6GG26	50S ribosomal protein L35	*rpmI*	2.51	0.0308	1.51	CC BP MF
Q99S51	50S ribosomal protein L13	*rplM*	2.49	0.0019	2.72	CC BP MF
Q99UI1	tRNA-2-methylthio-N(6)-dimethylallyladenosine synthase	*miaB*	2.42	0.0002	3.77	CC BP MF
Q6GEI3	50S ribosomal protein L3	*rplC*	2.33	0.0012	2.92	CC BP MF
Q6GEJ1	50S ribosomal protein L29	*rpmC*	2.32	0.0002	3.65	CC BP MF
Q2G222	N-acetylmuramoyl-L-alanine amidase domain-containing protein SAOUHSC_02979	*SAOUHSC 02979*	2.29	0.0074	2.13	CC MF
Q99RX9	Urease accessory protein UreG	*ureG*	2.24	0.0128	1.89	CC BP MF
Q6GG92	Uridine kinase	*udk*	2.17	0.0230	1.64	CC BP MF
Q6GHV8	50S ribosomal protein L32	*rpmF*	2.14	0.0002	3.63	CC BP MF
Q6GF03	Alanine racemase 1	*alr1*	2.11	0.0291	1.54	BP MF
Q99R06	HTH-type transcriptional regulator ArcR	*arcR*	2.09	0.0030	2.52	CC BP MF
Q99S93	UPF0457 protein SA1975.1	*SA1975.1*	2.08	0.0005	3.26	/
Q8NXX2	GTP cyclohydrolase FolE2	*folE2*	2.07	0.0001	3.93	BP MF
Q6GG25	Translation initiation factor IF-3	*infC*	2.04	0.0002	3.69	CC MF
Q6GEK8	30S ribosomal protein S11	*rpsK*	2.01	0.0008	3.08	CC BP MF
Q6GH63	UPF0154 protein SAR1353	*SAR1353*	2.00	0.0126	1.90	CC
Q6GET9	Pyrimidine-nucleoside phosphorylase	*pdp*	0.50	0.0011	2.96	BP MF
Q8NWB4	Endoribonuclease YbeY	*ybeY*	0.50	0.0300	1.52	CC BP MF
Q6GHQ7	Transcriptional regulator MraZ	*mraZ*	0.50	0.0000	4.33	CC MF
Q6GG19	Glyceraldehyde-3-phosphate dehydrogenase 2	*gapA2*	0.48	0.0013	2.88	CC BP MF
Q6GH55	Aconitate hydratase A	*acnA*	0.48	0.0003	3.59	BP MF
Q99TK9	GTPase Obg	*obg*	0.48	0.0005	3.26	CC BP MF
Q6GGG2	Aminomethyltransferase	*gcvT*	0.47	0.0035	2.45	BP MF
Q6GEC9	Putative 2-hydroxyacid dehydrogenase SAR2389	*SAR2389*	0.47	0.0003	3.56	MF
Q6GJR0	UPF0355 protein MRSA252	*SAR0405*	0.47	0.0023	2.64	/

Q6GFF6	Aspartyl/glutamyl-tRNA(Asn/Gln) amidotransferase subunit C	*gatC*	0.47	0.0065	2.19	BP MF
Q2YUL7	Pyrimidine-nucleoside phosphorylase	*pdp*	0.46	0.0009	3.03	BP MF
Q6GHG4	tRNA pseudouridine synthase B	*truB*	0.45	0.0017	2.77	BP MF
Q99V77	Putative phosphoesterase SAV1015	*SAV1015*	0.45	0.0054	2.27	MF
Q99UZ6	UPF0637 protein SA0957	*SA0957*	0.45	0.0308	1.51	/
Q99SZ3	Bacterial non-heme ferritin	*ftnA*	0.44	0.0019	2.72	CC BP MF
Q6GEU7	Probable DNA-directed RNA polymerase subunit delta	*rpoE*	0.44	0.0007	3.18	BP MF
Q6GDD3	Lipase 1	*lip1*	0.44	0.0032	2.50	CC BP MF
Q6GHQ1	Cell division protein DivIB	*divIB*	0.44	0.0201	1.70	CC BP
Q6GGY4	Peptide methionine sulfoxide reductase MsrB	*msrB*	0.44	0.0081	2.09	BP MF
Q6GJB5	Uncharacterized epimerase/dehydratase SAR0558	*SAR0558*	0.44	0.0006	3.21	MF
Q6GDV6	Uncharacterized oxidoreductase SAR2567	*SAR2567*	0.43	0.0015	2.82	MF
Q6GGZ5	2-oxoglutarate dehydrogenase E1 component	*odhA*	0.43	0.0032	2.50	BP MF
Q6GGY5	PTS system glucose-specific EIIA component	*crr*	0.42	0.0001	3.96	CC BP MF
Q99U83	Acylphosphatase	*acyP*	0.42	0.0050	2.30	MF
Q6GGT1	Elastin-binding protein EbpS	*ebpS*	0.40	0.0000	4.37	CC
P99174	Deoxyribose-phosphate aldolase 2	*deoC2*	0.39	0.0001	4.20	CC BP MF
Q99V08	UPF0356 protein SA0941	*SA0941*	0.39	0.0006	3.23	/
Q8NUR2	1-pyrroline-5-carboxylate dehydrogenase	*rocA*	0.39	0.0012	2.91	BP MF
Q6GH41	Probable tautomerase SAR1376	*SAR1376*	0.39	0.0109	1.96	BP MF
Q99TF3	Putative universal stress protein SAV1710	*SAV1710*	0.39	0.0002	3.63	CC
Q6GF07	Anti-sigma-B factor antagonist	*rsbV*	0.39	0.0106	1.98	BP MF
Q6GDQ0	ATP-dependent Clp protease ATP-binding subunit ClpL	*clpL*	0.38	0.0001	3.93	MF
Q6GII3	Glycine cleavage system H protein	*gcvH*	0.37	0.0012	2.92	CC BP

Q2G1C2	Teichoic acid ribitol-phosphate polymerase TarK	*tarK*	0.37	0.0224	1.65	CC BP MF
Q6GEA1	Formimidoylglutamase	*hutG*	0.36	0.0045	2.35	BP MF
Q6GJM8	Uncharacterized lipoprotein SAR0445	*SAR0445*	0.36	0.0403	1.39	CC
Q6GGZ6	Dihydrolipoyllysine-residue succinyltransferase component of 2-oxoglutarate dehydrogenase complex	*odhB*	0.33	0.0001	4.00	CC BP MF
Q6GEC4	Putative formate dehydrogenase SAR2393	*SAR2393*	0.33	0.0003	3.60	BP MF
Q6GHU0	Thioredoxin	*trxA*	0.33	0.0000	4.53	CC BP MF
Q99T01	UPF0435 protein SAV1880	*SAV1880*	0.33	0.0024	2.61	/
Q99S65	Uncharacterized hydrolase SAV2204	*SAV2204*	0.33	0.0024	2.63	MF
Q6GH72	Catalase	*katA*	0.31	0.0002	3.63	BP MF
Q6GDM0	Uncharacterized hydrolase SAR2661	*SAR2661*	0.27	0.0003	3.53	MF
Q6GE66	Probable malate:quinone oxidoreductase 1	*mqo1*	0.26	0.0015	2.83	BP MF
Q6GAW9	Ornithine aminotransferase 2	*rocD2*	0.26	0.0011	2.94	CC BP MF
Q6GF50	Uncharacterized leukocidin-like protein 1	*SAR2107*	0.25	0.0054	2.27	CC BP
Q99UC6	Uncharacterized protein SAV1352	*SAV1352*	0.25	0.0002	3.63	/
Q6GIF4	D-alanyl carrier protein	*dltC*	0.23	0.0038	2.42	CC BP MF
Q6GH10	Uncharacterized hydrolase SAR1410	*SAR1410*	0.22	0.0060	2.22	MF
Q5HEI1	Phospholipase C	*hlb*	0.22	0.0003	3.55	CC BP MF
Q6GE63	Uncharacterized lipoprotein SAR2457	*SAR2457*	0.20	0.0001	4.23	CC
Q6GF49	Uncharacterized leukocidin-like protein 2	*SAR2108*	0.19	0.0016	2.79	CC BP
Q8NVL8	Uncharacterized leukocidin-like protein 2	*MW1942*	0.18	0.0033	2.48	CC BP
Q6GGH6	Exodeoxyribonuclease 7 small subunit	*xseB*	0.16	0.0004	3.39	CC BP MF
Q6GIC7	Argininosuccinate synthase	*argG*	0.15	0.0001	4.16	CC BP MF
Q8NXF3	Argininosuccinate lyase	*argH*	0.14	0.0001	3.95	CC BP MF

Q6GE13	Gamma-hemolysin component C	*hlgC*	0.10	0.0017	2.77	CC BP MF
Q6GKB4	PTS system EIIBC component SAR0193	*SAR0193*	0.10	0.0001	4.09	CC BP MF
Q6GFB8	Protein map	*map*	0.10	0.0014	2.86	/
Q6GF48	Probable succinyl-diaminopimelate desuccinylase	*dapE*	0.09	0.0008	3.11	BP MF
Q6GE12	Gamma-hemolysin component B	*hlgB*	0.08	0.0014	2.84	CC BP MF
Q9EZ10	2,3,4,5-tetrahydropyridine-2,6-dicarboxylate N-acetyltransferase	*dapH*	0.07	0.0001	3.83	BP MF
Q2FWX9	4,4-diaponeurosporen-aldehyde dehydrogenase	*aldH1*	0.06	0.0349	1.46	BP MF
Q6GEA4	Urocanate hydratase	*hutU*	0.06	0.0023	2.64	CC BP MF
Q2G1X0	Alpha-hemolysin	*hly*	0.06	0.0007	3.15	CC BP MF
Q6GJZ6	Lipase 2	*lip2*	0.05	0.0005	3.27	CC BP MF
Q6GE14	Gamma-hemolysin component A	*hlgA*	0.03	0.0005	3.29	CC BP MF
Q99Q02	Regulatory protein MsrR	*msrR*	+	-		CC
Q8NVG5	Thymidine kinase	*tdk*	+	-		CC BP MF
Q6GJA6	Bone sialoprotein-binding protein	*bbp*	+	-		CC BP
Q8NXL2	SsrA-binding protein	*smpB*	+	-		CC BP MF
Q99R88	ATP-dependent Clp protease ATP-binding subunit ClpL	*clpL*	+	-		MF
Q8NXI0	3-dehydroquinate dehydratase	*aroD*	-	-		BP MF
Q99R80	Copper-exporting P-type ATPase	*copA*	-	-		CC BP MF
Q2YWL4	Extracellular matrix protein-binding protein emp	*emp*	-	-		CC
Q8NYC2	Lipase 2	*lip2*	-	-		CC BP MF
Q9RQL3	Peroxide-responsive repressor PerR	*perR*	-	-		CC MF
Q99X30	N-acetylmuramic acid 6-phosphate etherase	*murQ*	-	-		BP MF
Q99XA5	HTH-type transcriptional regulator NorG	*norG*	-	-		BP MF
Q2FZ95	Cell division protein FtsL	*ftsL*	-	-		CC BP

Q6GET8	Deoxyribose-phosphate aldolase 2	*deoC2*	-	-	CC BP MF
Q6GFT6	6,7-dimethyl-8-ribityllumazine synthase	*ribH*	-	-	CC BP MF
Q6GID1	Ornithine aminotransferase 2	*rocD2*	-	-	CC BP MF
Q6GIK4	Clumping factor A	*clfA*	-	-	CC BP
Q84BP4	Uncharacterized chromosomal cassette SCCmec type IVc protein CR006	CR006	-	-	/

[1] Up-regulated enzymes are highlighted in green; down-regulated enzymes are highlighted in red.
"+" stands for only detected in treatment group and "-" stands for only detected in control group.

Table S2. The differently changed metabolites in treatment group and control group.

Description	VIP	Fold change	p-value
Trimethylamine N-oxide	3.8523	0.0170	<0.0001
Pindone	1.7780	0.0232	<0.0001
2-Methylbutyroylcarnitine	1.1304	0.0445	0.0005
DL-3-Phenyllactic acid	4.6898	0.0479	0.0006
Acetylcarnitine	1.8241	0.0503	<0.0001
Hydroxyproline	5.9625	0.0637	<0.0001
Pantothenate	1.3736	0.0712	<0.0001
DL-2-Aminoadipic acid	1.0256	0.0788	<0.0001
Ala-Glu	4.3004	0.1001	<0.0001
Lys-Thr	1.2935	0.1008	0.0006
N-Carbamylglutamate	1.3385	0.1048	<0.0001
Phosphorylcholine	1.6875	0.1186	<0.0001
5-L-Glutamyl-L-alanine	1.3805	0.1214	<0.0001
Arg-Ala	2.4770	0.1219	<0.0001
Prazosin	1.7054	0.1277	<0.0001
5,2'-O-dimethylcytidine	1.2103	0.1339	<0.0001
Hydroxyphenyllactic acid	2.1231	0.1386	<0.0001
Heneicosanoic acid	1.1308	0.1455	0.0001
trans-Vaccenic acid	1.5032	0.1482	0.0034
Pro-Val	1.1899	0.1486	<0.0001
Ornithine	2.6732	0.1502	0.0039
N6-Acetyl-L-lysine	4.6505	0.1571	<0.0001
Behenic acid	1.6457	0.1720	0.0002
Pro-Tyr	1.0082	0.1729	<0.0001
Isobutyric acid	3.5791	0.1776	<0.0001
D-Mannose-6-phosphate	1.3535	0.1831	<0.0001
alpha-D-Glucose 1-phosphate	2.7328	0.1905	<0.0001
N-Acetylcadaverine	1.9814	0.1926	<0.0001
gamma-L-Glutamyl-L-glutamic acid	1.3239	0.1934	<0.0001
N2-Acetyl-L-ornithine	1.3643	0.1947	<0.0001
Anthranilic acid (Vitamin L1)	1.9361	0.1993	<0.0001
D-Proline	3.4006	0.2034	<0.0001
L-Pipecolic acid	1.9218	0.2090	<0.0001
N6-Methyladenine	2.3991	0.2113	<0.0001
Acetyl-DL-Valine	1.2270	0.2266	0.0003
N-Acetyl-L-tyrosine	1.1714	0.2329	<0.0001
Alpha-D-Glucose	9.6160	0.2432	<0.0001
Taurine	2.6777	0.2450	<0.0001
N-Acetyl-L-phenylalanine	1.9122	0.2485	0.0002
L-Citrulline	2.4971	0.2495	0.0040
N-Acetyl-L-alanine	1.7352	0.2505	<0.0001
Tyramine	1.2620	0.2525	<0.0001

N.alpha.-Acetyl-L-lysine	2.2759	0.2626	<0.0001
.gamma.-L-Glu-.epsilon.-L-Lys	1.7785	0.2645	<0.0001
Homocitrate	1.1816	0.2690	<0.0001
Lanosterol	1.0216	0.2719	<0.0001
2-Methyl-3-hydroxybutyric acid	1.3931	0.2868	<0.0001
Maleamic acid	1.3274	0.2904	<0.0001
N-Acetyl-L-glutamate	2.4218	0.2981	<0.0001
NG,NG-dimethyl-L-arginine(ADMA)	2.1959	0.3013	0.0014
Glycerol 3-phosphate	1.4822	0.3116	0.0001
L-Alanine	2.3976	0.3156	<0.0001
Nicotinate	1.6871	0.3169	<0.0001
D-Aspartic acid	1.8181	0.3263	<0.0001
L-Aspartate	3.4757	0.3295	<0.0001
Ramipril	1.8177	0.3323	<0.0001
Erucamide	2.2518	0.3410	<0.0001
Choline	2.4428	0.3423	0.0079
Pro-Ser	1.3026	0.3497	<0.0001
L-Carnosine	1.2546	0.3571	<0.0001
(S)-2-Hydroxyglutarate	2.0847	0.3761	<0.0001
Maleic acid	1.9868	0.3998	<0.0001
L-Methionine	2.4026	0.4024	<0.0001
Dimethylaminopurine	1.9606	0.4138	<0.0001
Phosphoenolpyruvate	2.9073	0.4335	<0.0001
Citraconic acid	1.0568	0.4389	<0.0001
Hydroxyisocaproic acid	1.8008	0.4437	0.0160
Lys-Leu	1.2875	0.4665	0.0070
DL-lactate	1.8060	0.4708	<0.0001
Creatinine	2.4564	0.4824	0.0002
Stearic acid	1.4430	0.5111	0.0002
1-Aminocyclopropanecarboxylic acid	1.2809	0.5322	<0.0001
Phytanic acid	4.5511	0.5389	0.0004
cis-9-Palmitoleic acid	2.0613	0.5391	0.0021
L-Lysine	2.0807	0.5445	0.0001
Pro-Asn	1.6176	0.5480	0.0005
L-Glutamate	5.2585	0.5559	<0.0001
DL-2-Phosphoglycerate	2.2993	0.5625	0.0434
Pro-Gln	1.0852	0.5644	0.0003
Hypoxanthine	7.5394	0.5646	<0.0001
Succinate	4.0659	0.5733	<0.0001
N-.alpha.-Acetyl-L-arginine	3.5649	0.5763	0.0100
Stearidonic Acid	1.4651	0.5924	<0.0001
Hypoxanthine	6.9866	0.6172	<0.0001
N-Acetyl-L-aspartic acid	1.4421	0.6364	0.0004
N-Formylmethionine	1.7229	0.6390	0.0037

Dimethylglycine	1.9240	0.6693	0.0021
L-Phenylalanine	3.5014	0.6761	0.0005
L-Tyrosine	1.5196	0.6976	0.0021
O-Succinyl-L-homoserine	1.0095	0.7400	0.0144
L-Carnitine	5.9866	0.7527	<0.0001
Glucosamine	1.0597	1.1405	0.0474
2-Hydroxyadenine	2.2711	1.2150	0.0356
D-Alanyl-D-alanine (D-Ala-D-Ala)	1.3194	1.2351	0.0098
2-Oxoadipic acid	6.3069	1.3764	0.0001
Triethanolamine	1.0469	1.4767	0.0411
Betaine	1.6157	1.5047	0.0194
Argininosuccinic acid	1.2873	1.6419	0.0086
S-Adenosylmethionine	2.2262	1.6948	0.0177
Cyclic adenosine diphosphate ribose	1.4640	1.7876	<0.0001
Beta-D-Fructose 6-phosphate	1.2265	1.8932	0.0159
Adenine	3.0591	2.0464	0.0002
L-Pyroglutamic acid	1.7447	2.1746	<0.0001
S-Methyl-5'-thioadenosine	17.3570	2.2053	<0.0001
Adenosine 5'-diphosphate (ADP)	1.3995	2.3064	<0.0001
L-Asparagine	1.0719	2.4099	0.0003
Adenosine	1.4782	2.4145	0.0369
Ribothymidine	1.2161	2.4950	<0.0001
2'-Deoxyadenosine 5'-monophosphate (dAMP)	1.2310	2.7339	0.0010
Uridine 5'-diphosphate (UDP)	1.1947	2.8391	<0.0001
Thymine	1.3910	2.8526	0.0011
Mevalonic acid	2.5257	2.8649	0.0001
Thymidine	1.0858	2.8700	0.0032
UDP-N-acetylglucosamine	2.6152	2.8919	<0.0001
L-Leucine	1.5705	2.9479	0.0057
Norharmane	4.2170	2.9509	<0.0001
.beta.-Homoproline	2.0018	3.0228	<0.0001
Adenosine monophosphate (AMP)	9.4155	3.1133	0.0001
Citrate	5.4237	3.1494	0.0006
Isovalerylglycine	1.0619	3.7104	0.0001
3'-Phosphoadenosine 5'-phosphosulfate (PAPS)	1.3034	3.7300	<0.0001
Ser-Val	1.0838	3.7585	0.0024
L-Isoleucine	1.5241	3.8388	0.0001
1-Methyladenosine	1.2276	3.8565	<0.0001
Xanthylic acid (XMP)	1.7562	3.8876	0.0001
ADP-ribose	1.4706	3.8954	<0.0001
Nicotinamide	3.2265	3.9223	<0.0001
Ala-Gly	1.0373	3.9781	<0.0001
cis-Aconitate	1.0689	3.9952	0.0059
Deoxyadenosine	1.3008	4.0581	0.0001

L-Valine	2.4293	4.0753	0.0003
Xanthosine	2.7263	4.1229	0.0095
Uridine 5'-triphosphate (UTP)	1.5235	4.2051	<0.0001
Deoxythymidine 5'-diphosphate (dTDP)	1.4589	4.2088	<0.0001
Thiamine monophosphate	2.9334	4.2994	0.0005
Nicotinamide adenine dinucleotide phosphate (NADP)	3.8567	4.3405	<0.0001
1-Palmitoylglycerol	1.1483	4.3785	0.0004
Xanthine	1.6188	4.7821	0.0017
alpha-N-Acetyl-L-glutamine	1.9803	4.7847	<0.0001
N-Acetyl-D-Glucosamine 6-Phosphate	1.1184	4.8295	0.0364
Nicotinamide adenine dinucleotide (NAD)	16.0191	4.9728	<0.0001
UDP-N-acetylmuraminate	2.3995	5.2603	<0.0001
Reduced nicotinamide adenine dinucleotide (NADH)	1.5505	5.3086	<0.0001
Guanosine 5'-monophosphate (GMP)	2.1654	5.6896	0.0005
Uracil	5.1152	5.7269	<0.0001
Pro-Asp	1.2089	5.8538	0.0005
Uridine diphosphate glucose(UDP-D-Glucose)	1.0770	6.4606	<0.0001
O-Phospho-L-homoserine	1.0729	6.4950	0.0002
D-Ribose 5-phosphate	1.2910	6.6718	<0.0001
Garcinol	1.6049	6.7089	<0.0001
Deoxyinosine	3.2675	6.9242	<0.0001
ADP-ribose	2.8948	7.1588	<0.0001
D-Ribulose 5-phosphate	1.8377	7.4642	<0.0001
L-Tryptophan	1.1758	7.5939	0.0001
Uridine 5'-monophosphate (UMP)	4.1288	7.6927	<0.0001
Glu-Lys	1.6766	7.8257	0.0009
Reduced nicotinamide adenine dinucleotide phosphate (NADPH)	1.3222	7.8762	<0.0001
Uridine	1.1117	8.1663	0.0002
Ser-Ile	1.2821	8.2446	0.0029
Cytidine 5'-monophosphate (CMP)	2.3203	8.3579	<0.0001
Glycerol 1-myristate	1.6339	8.9694	<0.0001
L-Leucine	6.5315	9.4795	0.0008
Deoxyadenosine	14.7852	9.8412	<0.0001
Urocanic acid	1.2113	10.0135	<0.0001
Adrenic Acid	1.5248	10.2491	<0.0001
Cytidine 5'-diphosphate (CDP)	2.7453	10.4083	<0.0001
Guanosine	3.6831	10.9055	<0.0001
Ser-Thr	1.0018	11.0811	0.0003
Inosine	1.5510	11.6406	<0.0001
L-Arginine	11.5877	12.1060	0.0041
3-Methylbutanoyl-CoA	3.1843	12.3029	<0.0001
Trehalose	1.8040	13.2911	<0.0001
Pristanic acid	4.3349	13.4636	<0.0001

Butyryl-coenzyme A (Butyryl-CoA)	3.7160	14.5221	<0.0001
Isobutyryl-CoA	3.5606	14.5721	<0.0001
Cytidine 5'-monophosphate	8.8744	15.6586	<0.0001
Cytidine 2',3'-cyclic phosphate	1.1238	17.3100	<0.0001
L-Kynurenine	1.0805	17.8993	0.0004
Galactinol	2.4616	19.0879	<0.0001
Guanosine	2.9907	19.2913	0.0001
Acetyl coenzyme A (Acetyl-CoA)	3.5325	22.2889	<0.0001
3',5'-Cyclic guanosine monophosphate	1.8732	29.2338	<0.0001
D-Arabinono-1,4-lactone	4.7785	30.1089	<0.0001
Coenzyme A (CoA)	1.5041	35.6922	0.0015
Diadenosine triphosphate	2.6785	36.1553	<0.0001
Adenosine 2',3'-cyclic monophosphate	2.3695	36.7952	0.0008
Cytosine	4.5134	41.3676	<0.0001
Adenosine 3',5'-cyclic phosphate (cAMP)	4.4631	63.9810	0.0002
Pseudouridine	6.0921	142.6499	<0.0001
Cytidine	4.6576	991.7952	<0.0001
